# Anti-Paratyphoid Inoculation with Grave Phenomena

**Published:** 1916-11

**Authors:** 


**Anti-Paratyphoid Inoculation With Grave Phenomena.** Albert Levy, Le Prog. Med., Sept. 5, reports the case of a man aged 35, previously in good health who had had two series of anti-typhoid vaccinations and the first injection of anti-paratyphoid vaccine, without marked reaction, except that after the latter, he had had four or five diarrhoeic stools a day and had suffered from lack of appetite, but did not report this condition to the surgeon. The second anti-paratyphoid injection was given 10 days after the first. Within ten minutes, local pain in the arm occurred, with general tremors and weakness. During the night, intense rigors occurred and several diarrhoeic discharges, blueness of extremities, nose, ears, etc., weak pulse, temperature 40.2 (104.4 F) state of collapse. 10 C.C. of camphorated oil and 1 C.C. of 1:1000 adrenalin were administered hypodermatically. The pulse was 150 the day following the inoculation but gradually fell to about 80 but fluctuated and the circulatory power was not normal for 2 or 3 months. There was complete anuria the first day followed by albuminuria till the 6th day. The stools were bloody for 2 days. Patches of pallor, blueness and red tip of nose gave the patient the appearance of a clown. Numerous ecchymotic spots formed with labial herpes on the sixth day. Complete recovery ultimately occurred.

